# New smokeless moxibustion for knee osteoarthritis: A study protocol for a multicenter, single-blind, randomized controlled trial

**DOI:** 10.1097/MD.0000000000030700

**Published:** 2022-10-07

**Authors:** Lusheng Chen, Xiumei Ren, Fengxing Li, Haiping Deng, Guanghong Ding, Wei Yao, Ling Zhao, Xueyong Shen

**Affiliations:** a Shanghai Key Laboratory of Acupuncture Mechanism and Acupoint Function, Department of Aeronautics and Astronautics, Fudan University, Shanghai, China; b Shanghai Municipal Hospital of Traditional Chinese Medicine, Shanghai University of Traditional Chinese Medicine, Shanghai, China; c Acupuncture and Tuina School, Shanghai University of Traditional Chinese Medicine, Shanghai, China.

**Keywords:** knee osteoarthritis, pain, smokeless moxibustion, traditional Chinese medicine

## Abstract

Knee osteoarthritis (KOA) is a chronic inflammatory disease with high morbidity and disability. As the aging and obese population increase, so will the medical services for this disease. The purpose of this study is to compare the clinical efficacy of herbal activated carbon smokeless moxibustion and traditional moxibustion in the treatment of KOA and to determine the clinical efficacy of herbal activated carbon smokeless moxibustion in the treatment of KOA.

**Methods/design:**

This is a multicenter, two parallel-group, single-blind, randomized controlled trial. Eighty-eight subjects with KOA (Kellgren Lawrence grade II or III) will be recruited and randomly treated with smokeless moxibustion or traditional moxibustion in the ratio of 1:1. The smokeless moxibustion group will use plant herbal activated carbon smokeless moxa cone. The traditional moxibustion group will be treated with pure moxa cone. Subjects in both groups will receive treatment at the affected knee(s) at the acupuncture point ST35, EX-LE2, and EX-LE4. Subjects in both groups will receive 3 sessions per week of moxibustion for 4 weeks. The primary outcome are changes in the Western Ontario and McMaster Universities Osteoarthritis Index pain scores from baseline to week 24. Secondary outcomes include visual analog scale, 50 yards fast walking time, short-form heath survey 36, overall clinical efficacy evaluation, self-assessment of safety, treatment credibility and expectancy, and cytokines related to osteoarthritis in serum.

**Discussion:**

This randomized single-blind controlled trial takes traditional moxibustion as the control group to provide strict evidence for the clinical efficacy and safety of herbal activated carbon smokeless moxibustion in the treatment of KOA.

## 1. Introduction

Knee osteoarthritis (KOA) is a chronic inflammatory disease with high incidence rate, ranks 11th in the global disability diseases. With the aging of the world population and the aggravation of obesity, the demand for medical treatment of KOA has substantially increased.^[1]^ Knee trauma, cold and humid environment, bad living habits, age and obesity are the risk factors of knee arthritis.^[2–7]^

The guidelines published by the Osteoarthritis Research Society International in 2019 propose a combination of drug and non-drug treatment as a method of non-surgical treatment.^[8]^ The existing medications for inflammatory pain have obvious side effects. Currently, there is no therapeutic method of complete cure for osteoarthritis. The treatment is limited to the relief of symptoms, so as to improve the quality of life and activity.^[9–11]^ It is also reported that chondroitin and glucosamine can slow down the injury of knee cartilage, but cannot reduce the pain.^[12,13]^ The effect of hormone injection is similar to that of physical therapy in the short term.^[14]^

Effective intervention on KOA may reduce knee pain, prevent complications, and even help to reduce mortality.^[15]^ Traditional moxibustion is a widely used characteristic treatment technology of traditional Chinese medicine, which is often used to treat KOA.^[16–18]^ However traditional moxibustion has some defects like time-consuming and laborious, the flame is easy to cause ambustion, and the treatment room also has many requirements. In particular, the smoke generated in the combustion process is not conducive to the health of physicians and patients which to a certain extent restricts its clinical application. The mechanism of moxibustion is related to its warm effect and radiation effect. The previous study of our research group found that the combustion temperature of plant herbal activated carbon smokeless moxibustion is close to that of traditional moxibustion. Infrared radiation characteristics peak of two kinds of moxibustion are similar to that of human acupoints deducting blackbody radiation and adenosine triphosphate energy metabolism, and can resonate with the molecules in acupoints and body tissues to produce curative effect, providing the physiological physics basis that smokeless moxibustion can be used as an alternative material for traditional moxibustion. It has been reported that infrared radiation has effects on cellular adenosine triphosphate metabolism, cyclooxygenase concentration, and bone repair.^[19,20]^ The efficacy of herbal active carbon smokeless moxibustion in the treatment of KOA has not been evaluated in clinical trials. The hypothesis is that 3 sessions per week of 2 kinds of moxibustion will have the clinical effect on KOA.

## 2. Method

### 2.1. Study design

This is a non-inferiority randomized single-blind controlled clinical trial, which aims to compare the treatment of typical inflammatory pain (KOA) with herbal activated carbon smokeless moxibustion and traditional moxibustion. The protocol has been registered in the Chinese Clinical Trial Registry (No. ChiCTR2200057923) and will be implemented in accordance with the declaration of Helsinki. Eligible participants will be randomly divided into 2 groups (new smokeless moxibustion group or traditional moxibustion group), with a distribution ratio of 1:1. The whole study will include 4 weeks of treatment and an additional 20 weeks of follow-up. The patient will be informed: You will have a 1 in 2 possibilities of receiving smokeless moxibustion or traditional moxibustion. Prior to randomization, written consent should be obtained from each participant.

**Figure 1.**
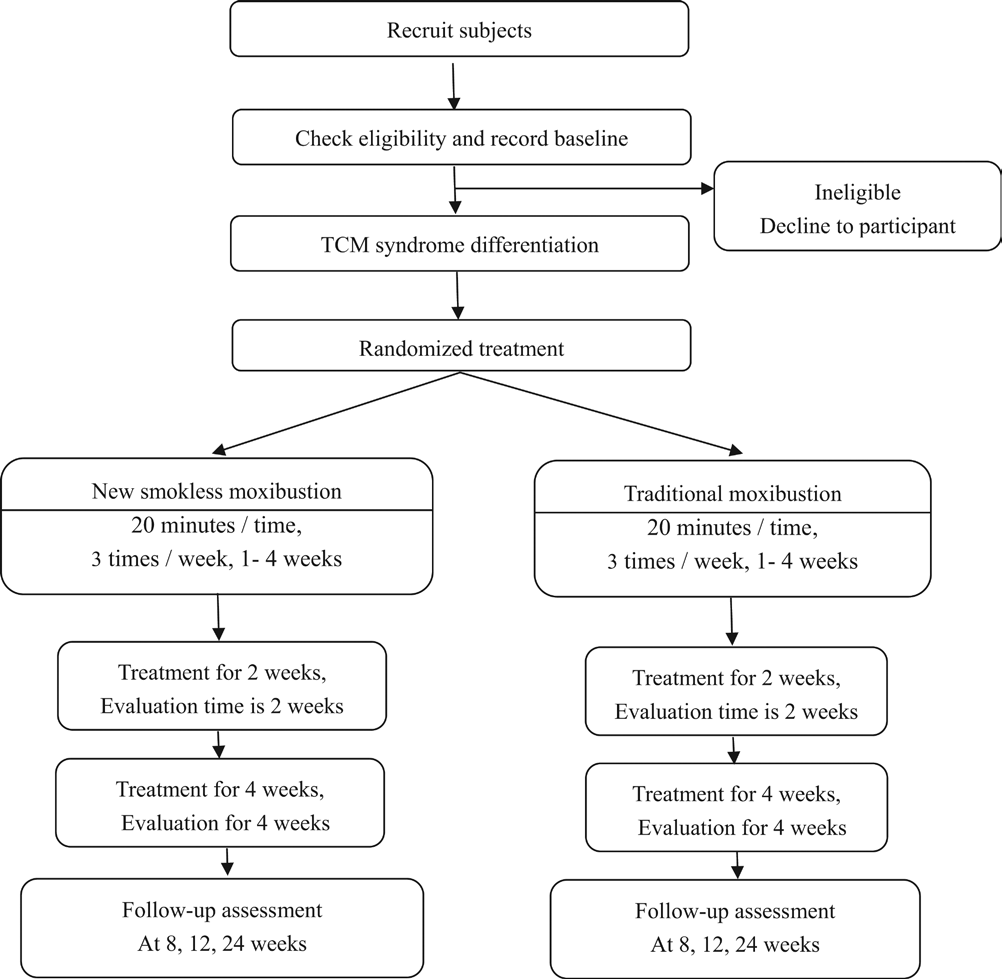
Study flow chart.

An overview of the study process is shown in Figure 1.

### 2.2. Study setting, recruitment, and ethics

The recruitment will be conducted in the outpatient department of acupuncture and moxibustion of Shanghai Municipal Hospital of Traditional Chinese Medicine Affiliated to Shanghai University of traditional Chinese medicine, Longhua Hospital Affiliated to Shanghai University of traditional Chinese medicine, and Shuguang Hospital Affiliated to Shanghai University of traditional Chinese medicine. A total of 88 subjects with KOA will be enrolled. This study has been approved by the medical ethics review committee of Shanghai Municipal Hospital of Traditional Chinese Medicine (No.2022SHL-KY-13). Recruitment will be carried out through social platforms, clinics, community publicity, etc. The investigators will contact those who may be willing to participate in this study by telephone, conduct preliminary screening according to the inclusion and exclusion criteria, and then arrange for a clinical physician to conduct a face-to-face baseline visit at an appropriate time, meanwhile performing radiological evaluation on subjects. The investigator will introduce the study protocol (i.e., study objective, procedure and time commitment, potential risks and benefits related to the study) to potential subjects in detail, and obtain written informed consent. The subject’s privacy information will be protected. Each subject will be given a unique random number as a direct identifier of the case report.

### 2.3. Inclusion criteria

1. Age 40 to 75 years.
2. According to the diagnostic criteria of KOA of the American rheumatology society.^[21–25]^
3. Radiographic confirmation of KOA (Kallgren Lawrence grade ≥ 1).^[26–28]^
4. Moderate or more severe knee pain for most of the past month; the subject’s visual analog scale baseline score for arthritic pain is 40 points and above.
5. Agree to accept the random assignment, understand, and be willing to sign the informed consent form.

### 2.4. Exclusion criteria

1. Knee pain caused by other diseases such as rheumatoid arthritis, fibromyalgia syndrome, chronic fatigue syndrome, and ankylosing spondylitis.
2. Received steroids in the past 3 months.
3. Received acupuncture treatment within 3 months.
4. Hyaluronate was injected into the joints in the past 6 months.
5. History of joint puncture or arthroscopy in the past year.
6. History of the knee/hip replacement surgery or planning to perform such surgery during the trial.
7. Use of other topical treatments, such as topical use of drugs.
8. Presence of any serious diseases including heart disease, lung disease, kidney disease, liver disease or malignant tumor, systemic infection or infectious disease, and mental illness.
9. Participation in another clinical study in the past 1 month.

### 2.5. Withdrawal criteria

Subjects who drop or discontinue prior to completion of the treatment period should conduct a questionnaire survey and those who have serious adverse reactions will be reported to the ethics committee.

1. Those who have not completed the treatment and have withdrawn before the end of treatment.
2. Any serious adverse reactions.
3. Major problems in the protocol design or trial process (such as those who are seriously infected due to blistering).

### 2.6. Randomization

A total of 88 subjects with KOA who are eligible per protocol will be randomly assigned to group A plant herbal activated carbon smokeless moxibustion group or group B traditional moxibustion group in a 1:1 ratio. After the investigator and the subject sign the informed consent, the investigator will perform a simple rheumatic examination. The investigator will make a diagnosis based on the knee X-ray film brought by the subject or the knee X-ray performed at the site. Only the subject who meet the inclusion/exclusion criteria will be randomized by a random number table. Then investigator will make a baseline assessment and arrange treatment.

### 2.7. Interventions

The treatment scheme of this experiment is based on the clinical treatment of KOA with traditional moxibustion, combined with blinding, considering the standardization requirements of the experimental design.

According to the literature review, the following 3 acupoints are selected as therapeutic acupoints. ST35, EX-LE2, and EX-LE4 are all located in the knee region, and they are common acupoints in the global clinical research of treating KOA.^[29]^ In anatomy, acupoints ST35 and EX-LE 4 are located close to the thinnest part of the knee joint, so the warm effect of moxibustion can directly reach the joint cavity. According to biomechanical study, most of the clinical diagnosis and treatment guidelines of KOA pay special attention to muscle strength training of quadriceps femoris.^[30–33]^ The muscle strength of the extensor and flexor groups of the knee joint is closely related to the symptoms of KOA.^[34]^ EX-LE2 is near the medial oblique and lateral femoral muscles, which can directly improve the state of the quadriceps femoris.

Subjects are allowed to take concomitant medication, but we will encourage them not to change their medications during the trial. However, other therapies such as acupuncture and sodium hyaluronate injection are not allowed.

The whole treatment process will be supervised by the staff of the study team. The two groups of subjects will be treated separately by strictly trained treatment operators.

### 2.8. New smokeless moxibustion group

The smokeless moxibustion group will a new smokeless moxa cone (1.5 * 2.0 cm, combustible for 20 minutes) produced by Jiangsu Sancai Wuyan Medical Technology Development Co., Ltd. will be used After the moxa cone is ignited, it is put into the moxibustion box and fixed on the acupoints. If the subject feels scorching hot, adjust the temperature by adjusting the height of the moxibustion box to avoid empyrosis.

### 2.9. Traditional moxibustion group

After the moxa cone is ignited, it is put into the moxibustion box and fixed on the acupoints. If the subject feels scorching hot, adjust the temperature by adjusting the height of the moxibustion box to avoid empyrosis.

Treatment of 2 groups will last for 20 minutes, 3 times a week, and altogether 12 times in 4 weeks (every Tuesday, Thursday, and Saturday). If the subject is unable to receive treatment on time, he or she will be required to complete the treatment within that week.

If any adverse event occurs, treatment will be suspended, and the investigator can decide whether to terminate the treatment.

### 2.10. Treatment blinding

Both smokeless moxibustion and traditional moxibustion are placed in an unmarked moxibustion box after ignition. Their appearance and operation procedures are the same. The combustion of moxa sticks in the box is invisible. The masked description used in the consent is as described in the “method/design and setting” above so that the subjects will be blinded until the completion of the study. The whole process is supervised by the coordinator of our study team in each center. All subjects in the study will be treated by trained operators. Due to the difference in the amount of smoke burned by the 2 kinds of moxa sticks, subjects of 2 groups will be treated in separate treatment rooms to avoid communication.

### 2.11. Outcomes

If the subject had a single knee joint pain, the outcome assessment will be evaluated for that knee. If both knees were affected and only one of them met the inclusion criteria, only the eligible knee will be evaluated. If both knees met the inclusion criteria, select the knee with more pain for evaluation.

### 2.12. Primary outcome indicators

The primary outcome measure is the change in pain relative to baseline at the Western Ontario and McMaster Universities Osteoarthritis Index (WOMAC) from baseline to week 24. The most painful knee joint for the subject will be recorded at the baseline, which will be the joint we measured throughout the trial. We will also measure WOMAC pain at week 2, 8, 12, and 24.

### 2.13. Secondary outcome indicators

The most important secondary outcome measurement is the improvement of WOMAC function. We will also assess the total score, function score, and stiffness score of WOMAC at baseline, week 2, 4, 8, 12, and 24. Visual analog scale pain score will be used to evaluate the arthritis pain of subjects at baseline, week 2, 4, 8, 12, 24. Short-form health survey 36 will be used to measure the quality of life of the subjects. In addition, we will evaluate the efficacy of a 5-point scale in the 4th week (1 = very poor, 2 = poor, 3 = general, 4 = OK, 5 = very good).

Any adverse event, whether related to treatment, requires the subject and investigator to report on each treatment or visit. Side effects included redness and blistering caused during treatment in both groups. Serious adverse events will be reported to the Ethics Committee. During the follow-up period (week 5–24), we will call the subject weekly to find out about the adverse reactions and side effects that may occur during the week.

After 4 weeks of treatment, the investigator will ask the subjects about the safety of the treatment including safe, fewer safe, safety problems, and serious safety problems. After each treatment, we will ask the subject’s feelings: “What do you feel during the treatment? 1. Heat; 2. Cold; 3. Pain; 4. no feeling; 5. Other, please describe.” The subjects will be allowed to take the analgesic or non-steroidal anti-inflammatory drugs used before the test, we will ask subjects to record the daily dose during the entire clinical trial, and then analyze the changes in the dose. We will measure the outcome of the baseline, week 2, 4, 8, and 12 at the investigation center. For the outcome of week 24, we will mail the questionnaire to the subjects and ask them to post them back after filling them out.

### 2.14. Exploratory outcome indicators

The exploratory outcome is the level of IL-1β, IL-2, MCP-1, cartilage oligomeric matrix protein (COMP), and tumor necrosis factor TNF-α in serum. Interleukin-1β is a key virulence factor for osteoarthritis.^[35]^ As a pro-inflammatory factor released by helper T cells, IL-2 stimulates the activation of relevant effector cells, resulting in the damage of synovium and cartilage in the joint.^[36]^ Studies have shown that higher levels of COMP are associated with higher severity of KOA, and vice versa indicating a lower degree of KOA.^[37,38]^ Increased levels of MCP-1 promote the conversion of monocytes into macrophages in the joint capsule,^[39]^ promote the development of an inflammatory response, and cause inflammatory damage to the joint capsule. The 5 mL fasting venous blood will be obtained before treatment and 4 weeks after treatment. After standing, the serum will be centrifuged, and the tubes will be placed in a refrigerator at 80°C for testing. The changes in the above markers in serum will be detected by enzyme-linked immunosorbent assay and Human Cytokine Array Kit. In order to reduce the influence of the circadian rhythm, each blood draw will take place between 10 and 11:30 in the morning. We will use the human cytokine array kit from R&D Systems to analyze osteoarthritis-associated cytokines in subjects’ serum. In addition to detecting the IL-1β, IL-2, MCP-1, and COMP, the kit can also detect additional 28 cytokines, so we can also explore whether other factors are involved in the pathogenesis of osteoarthritis.

### 2.15. Data collection and management

All of our measures are free of charge in order to ensure that subjects are fully followed. The case report form will be completed on paper and then entered into the spreadsheet. The School of Acupuncture and Massage, Shanghai University of Traditional Chinese Medicine will keep the original case report form and other forms (including informed consent forms). The required data will be collected according to the following case data collection schedule (Table 1). The Research Ethics Committee of Shanghai Municipal Hospital of Traditional Chinese Medicine will be independent of the investigators and the sponsor to audit the trial implementation every 6 months and decide whether to terminate the study early.

**Table 1:** Case data collection timetable.

	Before treatment	Week 2	Week 4	Week 8	Week 12	Week 24
Signing informed consent	√	—	—	—	—	—
Concomitant treatment	√	—	—	—	—	—
History of medication	√	—	—	—	—	—
Bilateral standing knee joint radiography	√	—	—	—	—	—
WOMAC	√	√	√	√	√	√
VAS	√	√	√	√	√	√
SF-36	√	—	√	—	√	√
Subject self-evaluation	—	—	√	—	√	—
Subject feeling during treatment (collected at each treatment)	√	√	√	—	—	—
Serum marker	√	—	√	—	—	—
Safety evaluation (collected each time you visit and follow up)	—	√	√	√	√	√
Other:						
Chinese medicine intake	√	√	√	√	√	√
Daily drug record (collected each time you visit and follow up)	√	√	√	√	√	√

SF-36 = short-form health survey; VAS = visual analog scale, WOMAC = Western Ontario and McMaster Universities Osteoarthritis Index.

### 2.16. Sample size

In the previous study of the study group, the mean value of pain improvement of WOMAC in patients with KOA in the traditional moxibustion group was 184.87. The 10% of the mean difference in pain improvement in the moxibustion group was used as the non-inferiority margin δ,^[40]^ that is, δ = 18.49, which was jointly determined by a statistician and a clinical expert engaged in KOA research. We used the non-inferiority test to calculate the sample size, α = 0.025, 1-β = 80%, which was calculated by the sample size estimation software PASS20.0.1 (PowerAnalyand SampleSize, PASS20NCSS), resulting in that 38 subjects were required in each group (see Fig. 1). In order to prevent dropouts leading to an inadequate sample size, a dropout rate of 15% was preset, and we would recruit 44 subjects per group, which required a total of 88 subjects to be recruited.

### 2.17. Statistical analysis

The intent-to-treat analysis is the principle of this study and includes all subjects in a given treatment arm as well as subjects who complete at least 1 treatment course. The level of significant difference is set at 0.05. Baseline characteristics will be summarized for both groups of subjects. For continuous results, the normal distribution of the data is labeled as the mean (standard deviation). The primary outcome measure is the effect value of the WOMAC pain score from baseline, using the *t* test to compare the smoke-free moxibustion group with the traditional moxibustion group. For secondary outcomes, unpaired scores including WOMAC Pain, Function, and Stiffness Scores, Short-Form health survey 36, Expectedness and Reliability of Treatment, and Overall Treatment Outcomes will be compared at all follow-up time points in both groups. Continuous variables will be compared using *t* test or Wilcoxon rank-sum test. We will use SPSS version 22 (IBM SPSS Statistics, New York, NY) for statistical analysis.

## 3. Discussion

The purpose of this study is to explore smokeless moxibustion as an effective moxibustion supplement and alternative therapy for the treatment of KOA. The trial will assess its non-inferiority compared to traditional moxibustion. Participants will receive 3 treatments per week. For external treatment of traditional Chinese medicine, the frequency of 3 times a week is the most commonly used method for the treatment of KOA. The use of single-blind randomized controlled trials in this trial can ensure the safety of subjects during the trial, and can also avoid the positive or negative effects of bias caused by subjective preferences for different therapies. This trial is a multicenter trial, which facilitates the collection of cases in a wide range in a short period of time, and the results are also more representative. This trial has some limitations. Due to the differences in the moxibustion materials used for smokeless moxibustion and traditional moxibustion, the operator will understand the grouping before placing the moxibustion box, which may lead to the operator’s basis. Therefore, operators will be rigorously trained in the assessment, treatment, follow-up, etc prior to the study. Traditional moxibustion produces strong smoke, which can be harmful to health,^[41]^ so many clinics and hospitals do not perform moxibustion therapy. New smoke-free moxibustion solves the problem of smoke produced by traditional moxibustion and also retains the warm effect, infrared characteristics, and unique odor of traditional moxibustion. Exploratory studies will help to determine the effect of new smokeless moxibustion and traditional moxibustion on serum parameters of KOA and explore objective parameters of clinical efficacy of KOA. This trial will provide reliable evidence for new smokeless moxibustion in the treatment of KOA and compensate for the lack of clinical efficacy research in this field.

## Author contributions

XYS and LZ designed the study as principal investigators. LSC, XMR, GHD, WY, HPD, and LZ participated in the design of the study and drafted the manuscript. LSC, XMR, HPD, and LZ are responsible for the recruitment and treatment of patients. All authors read and approved the final manuscript.

Writing – original draft: Lusheng Chen.

Writing – review & editing: Xiumei Ren, Fengxing Li, Haiping Deng, Guanghong Ding, Wei Yao, Ling Zhao, Xueyong Shen.
